# Predicting aggression in children with ADHD

**DOI:** 10.1186/1753-2000-8-15

**Published:** 2014-05-15

**Authors:** Elif Ercan, Eyüp Sabri Ercan, Hakan Atılgan, Bürge Kabukçu Başay, Taciser Uysal, Sevim Berrin İnci, Ülkü Akyol Ardıç

**Affiliations:** 1 Department of Psychological Counseling and Guidance, Ege University Faculty of Education, Izmir, Turkey; 2 Department of Child and Adolescent Psychiatry, Ege University Faculty of Medicine, Izmir, Turkey; 3 Department of Educational Sciences Measurement and Evaluation in Education, Ege University Faculty of Education, Izmir, Turkey; 4 Ege University Institute on Drug Abuse, Toxicology and Pharmaceutical Science, İzmir, Turkey; 5 Child and Adolescent Psychiatry, Denizli, Turkey

**Keywords:** Aggression, ADHD, Structural equation modeling

## Abstract

**Objective:**

The present study uses structural equation modeling of latent traits to examine the extent to which family factors, cognitive factors and perceptions of rejection in mother-child relations differentially correlate with aggression at home and at school.

**Methods:**

Data were collected from 476 school-age (7–15 years old) children with a diagnosis of ADHD who had previously shown different types of aggressive behavior, as well as from their parents and teachers. Structural equation modeling was used to examine the differential relationships between maternal rejection, family, cognitive factors and aggression in home and school settings.

**Results:**

Family factors influenced aggression reported at home (.68) and at school (.44); maternal rejection seems to be related to aggression at home (.21). Cognitive factors influenced aggression reported at school (.-05) and at home (-.12).

**Conclusions:**

Both genetic and environmental factors contribute to the development of aggressive behavior in ADHD. Identifying key risk factors will advance the development of appropriate clinical interventions and prevention strategies and will provide information to guide the targeting of resources to those children at highest risk.

## Background

ADHD is one of the most prevalent childhood disorders, and it is a community health problem that may result in significant psychiatric, social and academic problems if not treated. ADHD frequently co-occurs with other psychiatric disorders [1,2]. Research shows that aggression is an important associated feature of ADHD, and it is essential in understanding the impact of the disorder and its treatment [3]. The presence of comorbid aggression in ADHD does not appear to be spurious, and the severity and/or presence of aggression and ADHD may significantly impact its long-term prognosis. The etiology of aggression in ADHD is not clearly understood. However, aggression can be considered to be an outcome of the interaction between genetic and environmental factors [4]. Aggression is thought to be inherited, and the concordance of maternal twins is between .28 and .72 [5]. Compared to children who only have ADHD, it is more likely that children with ADHD and ODD or CD have fathers with an Antisocial Personality Disorder. Pfiffner et al. [6] found that children who have fathers with Antisocial Personality Disorder are more at risk for developing behavioral problems.

The most significant family factors influencing the occurrence of aggression in ADHD are as follows: large family size, the attitude of the family towards aggression, disciplinary or negative parenting, low socio economic status and family conflict [7]. Extended family and low socio economic status may cause aggression as a result of inadequate attention.

Parental attitudes are particularly important in psychiatric disorders, including aggression and ADHD [8]. However, there is a gap in the literature regarding the nature of the relationship between negative parental attitudes and psychiatric disorders that influence childhood aggression. The debate over whether aggression in children caused by parents’ lack of interest and/or their hostile and critical attitudes towards their children, or whether negative parenting is instead caused by children’s behavioral problems remains unresolved [9].

Cognitive deficits primarily in the verbal area play a role in the etiology of aggression. Previous data regarding the interaction between cognition and aggression reveal such general cognitive predictors of aggression as lower intelligence quotients, reading difficulties, and problems associated with attention and hyperactivity [10]. Many studies suggest that aggressive children experience problems in social cognitive areas [11,12] and have lower IQ scores [13,14]. In a meta-analysis of twenty-seven studies, seventeen studies reported negative associations between cognitive functions and disruptive behaviors [15].

Some of the most comprehensive research examining the relationship between ADHD and aggression using advanced statistical analyses has been conducted by Miller et al. [16]. In that study, 165 children with ADHD and disruptive behaviors between the ages of 7 and 11 were tested using structural equation modeling (SEM) to determine the influence of family and cognitive factors on aggression. One of the most important characteristics of the study is that it attempts to explain aggression in children with ADHD with information from two sources: parents and teachers. Family factors including present and past aggression by parents and the number of siblings are examined. Cognitive factors, verbal IQ, reading and mathematical achievement are also examined. The study found that family factors are related to aggression at home and at school, whereas cognitive factors are only related to aggression at school.

The purpose of our study is to evaluate the influence of family, parent–child relations and cognitive factors on the development of aggression in children within a larger and a non-western sample. We use structural equation modeling and include information from the parents, teachers and the child as the information source. This method is ideal, as it is important to receive information from multiple sources to explain a multicomponent concept such as aggression. Accordingly, we include evaluations of the mothers’ acceptance or rejection of the child with ADHD in the structural equation model in addition to information received from parents and teachers. To our knowledge, this is the first study to consider information from the parent, teacher and the child regarding aggression in ADHD. In addition, we examine mother-child relationships in detail regarding the etiology of aggression [8,16], as we consider it crucial to include the perception of acceptance or rejection of children with ADHD by their mothers as a possible latent factor.

In our study, past and current aggression by the parents, the number of people living in the home and the number of siblings were used as family factors. To define cognitive factors in the present study, verbal and performance IQ and school success variables are used. To evaluate the perceptions of children regarding their mothers’ acceptance or rejection, warmth, aggression and rejection variables specified in the theory of parental acceptance and rejection are used [17].

## Methods

### Diagnosis of ADHD

In total, 476 subjects referred to the Disruptive Behavior Disorders Clinic in 2011 with a diagnosis of ADHD with aggressive behaviors were included in the study, in addition to their parents and teachers. Approval from The Institutional Review Board (IRB) at the Ege University School of Medicine was attained before the study began, and informed consent was gathered from the parents.

Our recruitment and screening procedures were designed to collect data from a carefully diagnosed sample of children for ADHD comorbidities and subtypes. The children were first interviewed by a senior child psychiatry resident using the Schedule for Affective Disorders and Schizophrenia for School Age Children: Present and Lifetime version (K-SADS-PL) [18]. The K-SADS-PL is a highly reliable semi-structured interview for the assessment of a wide range of psychiatric disorders. Cognitive assessments were performed using the Wechsler Intelligence Scale for Children-Revised (WISC-R) [19]. Subjects with an IQ less than 70 were excluded from the study. Those who met the inclusion criteria for the study also completed the Children’s Aggression Scale-Parent and Teacher Versions (CAS-P, CAS-T), Teacher Report Form (TRF), Turgay DSM-IV Disruptive Behavior Disorders Rating Scale (T-DSM-IV-S) parent and teacher forms, and the Parental Acceptance and Rejection Questionnaire (PARQ), completed by both the parents and teachers of the participants.

The returned parent and teacher version of T-DSM-IV-S forms were scored, and the children who scored less than one standard deviation below the relevant age norms on the Attention Deficiency and Hyperactivity Disorder subscales were excluded from the study. The T-DSM-IV-S was developed by Turgay [20] and translated and adapted by Ercan, Amado, Somer, & Cikoglu [21]. The T-DSM-IV-S is based on DSM-IV diagnostic criteria and assesses hyperactivity-impulsivity (9 items), inattention (9 items), opposition-defiance (8 items), and conduct disorder (15 items). Symptoms are scored by assigning a severity estimate for each symptom on a 4-point Likert scale (0 = not at all; 1 = just a little; 2 = quite a bit; and 3 = very much). The subscale scores on the T-DSM-IV-S were calculated by summing the scores on the items of each subscale. Similar scales derived from the DSM-IV diagnostic criteria for AD/HD, such as the AD/HD Rating Scale IV, have been shown to have adequate criterion-related validity and good reliability in different cultures both by parents and teachers [22,23]. The second diagnostic interview was conducted by an experienced child psychiatrist who knew that the child was a candidate for the study but was blind to the first judge’s diagnosis of comorbid disorders and ADHD subtypes. “A best estimate procedure” was used to determine the final diagnoses. “Best estimate procedure” is defined here as determining the diagnostic status after reviewing all teacher and parent scales and the K-SADS-PL, and WISC-R results.

### Dependent variables of the study

This study has two main dependent measures: aggression at home and aggression at school in elementary school students with ADHD.

### Children’s aggression scale – parent & teacher forms (CAS-P & CAS-T)

These scales were designed by Halperin et al. [24,25]. Both the 33-item CAS–P and 23-item CAS–T require informants to indicate the frequency (i.e., never, once per month or less, once per week or less, 2–3 times per week, or most days) with which the child has engaged in various aggressive behaviors during the past year. The CAS–P was entered into the model to indicate aggression in the home, and the CAS–T was entered to indicate aggression in school settings. Each test has five separate subscales: verbal aggression, aggression against objects and animals, provoked physical aggression, initiated physical aggression, and the use of weapons.

### Independent variables of the study

This study includes three independent measures of familial risk factors, cognitive risk factors, and children’s perceptions of acceptance and rejection in their relationships with their mothers.

Familial risk factors were evaluated by interview. A child psychiatrist asked the parents about the number of siblings, the number of people living in the home, and the parents’ present and past history of aggression. The Teacher Report Form (TRF) was used to obtain the children’s academic performance, and the Wechsler Intelligence Scale for Children-Revised (WISC-R) was used to assess cognitive risk factors. The “Parental Acceptance/Rejection Questionnaire (PARQ)” was used to determine the children’s perceptions of their acceptance/rejection by their mothers.

### The Parental Acceptance/Rejection Questionnaire (PARQ)

This scale was designed by Rohner, Saavedra and Granum in 1978 to assess the perceived acceptance/rejection of children with respect to their relationships with their parents. The PARQ includes four sub-scales: “Warmth (20 items), Hostility/Aggression (15 items), Neglect and Indifference (15 items), and Undifferentiated Rejection (10 items)”. The total scores for these sub-scales reflect the degree of perception, with higher scores indicating perceived rejection.

### Teacher Report Form (TRF)

The Teacher Report Form (TRF) was developed by Achenbach and Edelbrock [26] and adapted by Erol, Arslan, & Akçakın [27]. The Turkish Form of the TRF is normed for children 4–18 years of age and provides reliable and valid measures of the children’s school adaptation and problematic behaviors.

### Statistical methodology

In the first part of the data analysis, we used IBM PASW Statistics 18 for descriptive statistical analyses, and the data were presented as means (standard deviations), percentages, medians, and minimum and maximum values, where appropriate. In the second part, we used SPSS AMOS 18 for testing the structural equation model.

## Results

In total, 476 subjects between 7 and 15 years of age (±2.11) diagnosed with ADHD were included in the study. The majority (79% of participants; n = 376) were boys, and 21% (n = 100) were girls. The distribution of diagnostic groups and their percentages in the study population are presented in Table 1. The cases were diagnosed as “pure” ADHD (37.8%), ADHD + ODD (44.3%) and ADHD + CD (17.9%). Descriptive statistics for the observed variables in the SEM hypothesis are presented in Table 2.

**Table 1:** **Diagnoses of participants and their percentages in the study population** (**N** = **476**)

**Diagnosic group**	**N**	**Percent**
ADHD	144	%37.8
ADHD + ODD	210	%44.3
ADHD + CD	85	%17.9
***TOTAL***	*476*	%*100*

*ADHD*: Attention Deficit Hyperactivity Disorder, *ADHD* + ODD: Attention Deficit Hyperactivity Disorder and Oppositional Defiant Disorder, *ADHD* + CD: Attention Deficit Hyperactivity Disorder and Conduct Disorder.

**Table 2:** **Descriptive statistics of observed variables in the SEM hypothesis** (***N*** = ***476***)

***Observed variables***	**Mean**	**SD**	**n**	**%**	**Median**	**Min**	**Max**
Warmth	31.79	12.87					
Aggression	25.71	9.13					
Neglect	22.49	7.37					
Rejection	17.03	6.01					
Aggression of Mom, Present			198	61.5%			
Aggression of Dad, Present			137	42.9%			
Aggression of Mom, Past			88	27.8%			
Aggression of Dad, Past			132	41.3%			
Number of people living in the home					4	2	9
Number of siblings					1	0	4
Verbal IQ	96.70	16.64					
Performance IQ	102.43	18.27					
School success	47.90	12.28					
Verbal aggression	11.59	9.88					
Aggression against objects	2.31	2.36					
Provoked aggression	4.97	4.51					
Initiated aggression	2.84	3.72					
Weapon use	0.05	0.31					
Verbal aggression	5.64	5.80					
Aggression against objects	1.51	2.59					
Provoked aggression	2.93	3.26					
Initiated aggression	2.11	2.75					
Weapon use	0.03	0.28					

**Figure 1:**
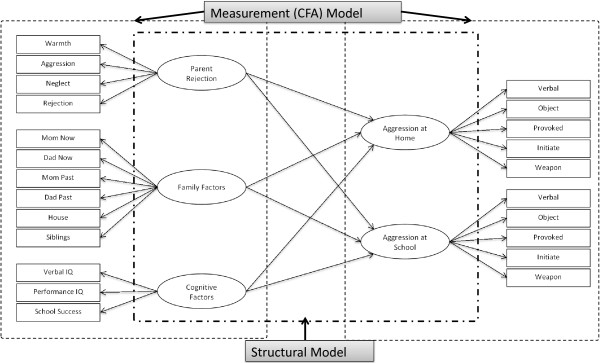
**Structural equation modeling of aggression in elementary school students with ADHD (*standardized solution; N*** **=** 476; *: *p* **<** *0.05*, **: *p* **<** ***0.001*).**

SEM analysis of our proposed model consisted of two separate elements, of which the first is a measurement model (confirmatory factor analysis-CFA) and the second is a structural model (Figure 1).

### Measurement model (confirmatory factor analysis)

The measurement model based upon a confirmatory factor analysis indicated that each of our measures was related to the latent variables with determination coefficients ranging from .92 to .01. Standardized and unstandardized regression weights, determination coefficients, and significance levels of these variables are shown in Table 3.

**Table 3:** **Unstandardized estimates**, **standardized estimates**, **determination coefficients**, **and significance levels for model in Figure **1 (**N** = **476**)

***Measurement *** **( *CFA *) *model***		**Unstandardized (S.E.)**	**Standardized**	***R***^**2**^	**p**
Parent Rejection	→Warmth	1	0.25	0.06	
	→Aggression	2.732 (0.68)	0.95	0.89	<0.001
	→Neglect	1.867 (0.47)	0.80	0.64	<0.001
	→Rejection	1.715 (0.43)	0.90	0.81	<0.001
Family Factors	→Aggression of Mom, Present	0.912 (0.32)	0.33	0.11	0.004
	→Aggression of dad, Present	0.706 (0.30)	0,25	0.06	0.020
	→Aggression of Mom, Past	0.261 (0.24)	0.10	0.01	0.285
	→Aggression of dad, Past	1	0.36	0.13	Na
	→Number of people living in the home	1.492 (0.38)	0.29	0.08	<0.001
	→Number of siblings	1	0.24	0.06	Na
Cognitive Factors	→Verbal IQ	3.344 (0.82)	0.88	0.78	<0.001
	→Performance IQ	2.883 (0.60)	0.70	0.49	<0.001
	→School success	1	0.36	0.13	Na
Aggression at Home	→Verbal aggression	5.474 (0.46)	0.87	0.75	<0.001
	→Aggression against objects	1	0.66	0.44	Na
	→Provoked aggression	2.292 (0.20)	0.79	0.63	<0.001
	→Initiated aggression	1.948 (0.17)	0.82	0.67	<0.001
	→Weapon use	0.035 (0.01)	0.18	0.03	0.006
Aggression at School	→Verbal aggression	2.535 (0.17)	0.86	0.75	<0.001
	→Aggression against objects	1	0.76	0.58	Na
	→Provoked aggression	1.582 (0.09)	0.96	0.92	<0.001
	→Initiated aggression	1.251 (0.08)	0.90	0.81	<0.001
	→Weapon use	0.035 (0.01)	0.24	0.06	<0.001
***Structural model***					
Parent rejection	→Aggression at Home	0.101 (0.04)	0.21		0.012
Parent rejection	→Aggression at School	0.051 (0.04)	0.08		0.238
Family Factors	→Aggression at Home	6.129 (1.82)	0.68		<0.001
Family Factors	→Aggression at School	4.959 (1.45)	0.44		<0.001
Cognitive Factors	→Aggression at Home	-0.043 (0.03)	-0.12		0.032
Cognitive Factors	→Aggression at School	-0.024 (0.03)	-0.05		0.028

χ^2^_(223)_ = 249.199, p = 0.11, CMIN/DF = 1.117, NFI = 0.906, CFI = 0.989, IFI = 0.989, TLI = 0.986, RMSEA = 0.019.

### Categorical variables

The dichotomous variables of our data were fathers’ or mothers’ presence of aggression whether at present or at past. Until recently, two primary approaches to the analysis of categorical data [28,29] have dominated this area of research. Both methodologies use standard estimates of polychoric and polyserial correlations, followed by a type of asymptotic distribution-free (ADF) methodology for the structured model. However, because of the ultra-restrictive assumptions of these methodologies, they are impractical and difficult to meet.

AMOS software uses Bayesian estimation (BE) method for categorical data via an algorithm termed the Markov Chain Monte Carlo (MCMC) algorithm.

Our data isn’t normally distributed so to estimate the parameters, the model is put in a Bayesian framework. After BE procedure we treated our categorical variables with a maximum likelihood (ML) procedure. The BE and ML procedures showed similar results with minimal or no differences. The comparisons of BE and ML results are shown in Table 4.

**Table 4:** **Comparison of factor loading unstandardized parameter estimates**: **maximum likelihood versus Bayesian estimation**

		**Estimation approach**	
***Measurement (CFA) model***		**ML**	**Bayesian**
Parent rejection	→Warmth	1	1
	→Aggression	2.732	2.75
	→Neglect	1.867	1.70
	→Rejection	1.715	1.73
Family Factors	→Aggression of Mom, Present	0.912	0.86
	→Aggression of Dad Present	0.706	0.65
	→Aggression of Mom, Past	0.261	0.26
	→Aggression of Dad Past	1	1
	→Number of people living in the home	1.492	1.48
	→Number of siblings	1	1
Cognitive Factors	→Verbal IQ	3.344	3.42
	→Performance IQ	2.883	2.65
	→School success	1	1
Aggression at Home	→Verbal aggression	5.474	5.65
	→Aggression against objects	1	1
	→Provoked aggression	2.292	2.15
	→Initiated aggression	1.948	1.93
	→Weapon use	0.035	0.04
Aggression at School	→Verbal aggression	2.535	2.40
	→Aggression against objects	1	1
	→Provoked aggression	1.582	1.66
	→Initiated aggression	1.251	1.25
	→Weapon use	0.035	0.03
***Structural model***			
Parent rejection	→Aggression at Home	0.101	0.11
Parent rejection	→Aggression at School	0.051	0.06
Family Factors	→Aggression at Home	6.129	6.13
Family Factors	→Aggression at School	4.959	4.59
Cognitive Factors	→Aggression at Home	-0.043	-0.03
Cognitive Factors	→Aggression at School	-0.024	-0.04

### Structural model

**Figure 2:**
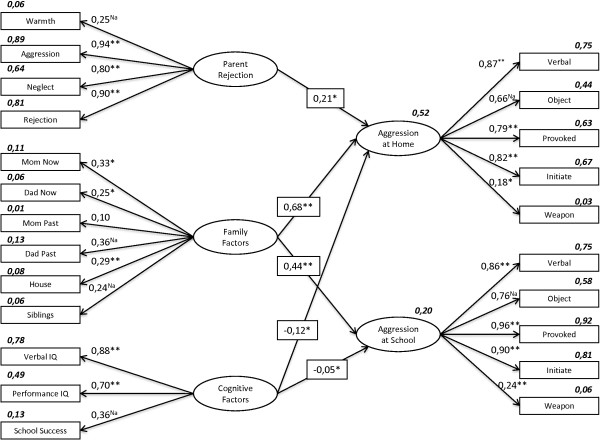
**Structural equation modeling of aggression in elementary school students with ADHD (*standardized solution*; *N*** **=** *476*; *: *p* **<** *0.05*, **: *p* **<** ***0.001*).**

In the second part of SEM analysis, we calculated estimates of the relationships, and we tested our model for fit. The structural model analysis in our study revealed statistically significant cross-loadings of aggression at home and aggression at school with the perception of acceptance/rejection by the mothers, family factors, and cognitive factors (Figure 2). There was a non-significant loading of the Perception of Acceptance or Rejection in Parent Relationships on aggression at school. The standardized and unstandardized regression weights and the significance levels of these variables are shown in Table 3.

### Testing the model-fit

The χ^2^ value of our model was 249.199, which is a large value. The Likelihood Ratio Test of the null hypothesis (H_0_) of this χ^2^ value revealed a non-significant probability, p = .11. As the χ^2^ probability of .11 was non-significant (p > .05), our model fit the data well.

The χ2 value of our model was 249.199, which is a large value. Because the χ2 statistic equals (N–1) F_min_, which means sample size minus 1, multiplied by the minimum fit function, this value tends to be substantial when the model does not hold and when sample size is large [30]. When our sample size, which is large enough, is considered, a higher χ2 value does make sense. The Likelihood Ratio Test results of the null hypothesis (H_0_) of this χ2 value revealed a non-significant probability, p = 0.11. The probability value associated with χ2 represents the likelihood of obtaining a χ2 value that exceeds the χ2 value when H_0_ is true. Thus, the higher the probability associated with χ2, the closer the fit between the hypothesized model (under H_0_) and the perfect fit [31]. As of our probability of 0.11 reveals (p > 0.05, non-significant), our model can be defined as a well-fitted model.

We used the CMIN/DF value as a second measure to test the fit of our model. Values of CMIN/DF lower than 2 indicate an acceptable fit [32-34], and our model fulfilled this criterion (CMIN/DF = 1.117).

The NFI value was .906, and the CFI value was .989 as shown in Table 3. The NFI value suggested that the model fit was only marginally adequate (NFI: .906), yet acceptable, but the CFI value suggests a superior fit (CFI: .989). The Incremental Index of Fit (IFI) [35] was developed to address issues of parsimony and sample size, which are known to be associated with the NFI. Unsurprisingly, our IFI of .989 is more consistent with the CFI and reflects a well-fitting model. Finally, the Tucker-Lewis Index (TLI) [36], consistent with the other indices noted here, yielded values ranging from zero to 1.00, with values close to .95 (for large samples) being indicative of good fit [37]. As shown in Table 3, our TLI value of .986 is indicative of a superior fit of our model.

The final index was the Root Mean Square Error of Approximation (RMSEA). This index was one of the most informative criteria in covariance structure modeling. The RMSEA takes into account the error of approximation in the population and asks the question “How well would the model, with unknown but optimally chosen parameter values, fit the population covariance matrix if it were available?” [38]. This discrepancy, as measured by the RMSEA, is expressed per degree of freedom, thus making it sensitive to the number of estimated parameters in the model (i.e., the complexity of the model); values less than .05 indicate good fit. The RMSEA value in our model was .019 as shown in Table 3, which represents a good fit.

When all of the indices are considered, we conclude that the proposed model fits our data well. The child’s perception of acceptance/rejection by the mothers significantly predicts aggression at home (β = .21, p = .012), whereas this perception does not predict aggression at school (p = .238). Family factors significantly predict aggression at home (β = .68, p < .001), and aggression at school (β = .44, p < .001). Likewise, cognitive factors significantly predict aggression at home (β = -.12, p = .032) and aggression at school (β = -.05, p = .028).

When all predictors of aggression levels are considered together, they predict 52% of the variance in overall aggression at home and 20% of the overall variance in aggression at school.

## Discussion

Even though aggressive behavior in children with ADHD is highly prevalent, it is not well understood [3]. Despite the existing literature on the influence of family factors, cognitive function and parent–child relationship problems on aggression in ADHD, there are few studies concerning the relationships of these factors with aggression at home and school. To the best of our knowledge, this is the first study examining the influence of family, cognitive and maternal acceptance or rejection factors on school-age children with ADHD with a large sample and using structural equation modeling.

The most important finding from this study is that family is the most important factor in predicting aggression in children with ADHD both at school and at home. This finding is in accordance with the findings of Miller et al. [16], which also model factors relating to aggression in ADHD with similar methodologies and statistics [16]. In both studies, family factors are found to be the most important factors in aggression both at school and at home. In our study, parents’ past and present aggression, the number of siblings and the number of people living in the same home are also evaluated as potential family indicators. We find that the number of siblings and the number of people living in the home do not significantly predict aggression at school or at home. Parents’ past and present aggression is the most important variable for predicting the aggression of children at school and at home. This finding is consistent with previous research, which clearly suggests that parents’ antisocial behavior is strongly and specifically related to their children’s aggressive behavior [39]. Although it is difficult to parse out the genetic and environmental influences, it is likely that aggressive parents play an important role in the emergence and persistence of aggression in children. For example, one study indicates that the more the aggressive parent is absent from the home, the smaller the effect that parent’s behavior has on the behavior of the children in the home [40]. Even if the genetic contribution of parents’ aggressive behavior is controlled, parental aggression nonetheless affects the child’s aggressive behaviors [41]. These findings in these studies support the importance of modeling environmental effects.

In our study, we evaluated the perceptions of children with ADHD regarding their acceptance or rejection by their mothers. The child’s perception of acceptance of rejection by the mothers is only related to aggression at home and not to aggression at school. In addition, we found that family factors predict aggression at home more than acceptance or rejection by the mother.

This finding suggests that the relationship between parenting and children’s behavior may be more complicated than previously thought, though it is in accordance with other studies of the influence of maternal attitudes on childhood aggression. In contrast with these previous studies, recent studies show that the correlation between parenting and children’s behavioral problems may not be linear. Yeh, Chen, Raine, Bakre, & Jacobson [42] find that the correlation between parenting and children’s behavioral problems depends upon the intensity of the children’s behavioral problems. In other words, similar parental attitudes may have different influences on different children. Cartwright et al. [43] also found that negative maternal emotions expressed towards children with ADHD (e.g., low warmth and hostility/criticism) are more damaging than emotions expressed towards children without ADHD. In this case, in addition to the impact of negative parenting on behavioral problems in children, it is important to also consider the influence of children’s behavioral problems on parents’ attitudes. In the study of Lifford et al. [44] a casual hypothesis of family relations influencing ADHD symptoms was not supported. Moreover, in many studies evaluating parental attitudes towards ADHD, parental attitudes improve after the administration of methylphenidate for the treatment of their children’s ADHD [45]. As a result of treatment, the resulting amelioration of the behavior may change the mother’s attitude towards the child. Based on these findings, the fact that maternal acceptance or rejection predicts childhood aggression only at home and is less predictive than other family factors suggests that parent–child relations have a secondary influence in cases of ADHD and that past and current parental aggression are the most important factors.

The third aim of our study was to evaluate the effects of cognitive factors on aggression in children with ADHD. Our findings reveal that children with lower cognitive function show more aggressive behaviors both at school and at home. This finding is consistent with many other studies in the literature, which also report that aggressive children have problems in social cognitive areas [10,11] and have lower IQ scores [12-14]. However, in our study, the correlation between cognitive factors and aggression at school and at home is less influential than family factors. This new information suggests that cognitive factors may have a limited scope of influence.

### Limitations

The most important limitation of this study is its cross-sectional methodology. Longitudinal studies are needed to better assess aggression in cases of ADHD. In addition, this study was not able to evaluate whether aggression is relational or social. The fact that the family’s socioeconomic situation was not assessed in detail is another limitation of our study. Another limitation of our study is that maternal acceptance and rejection perceptions were assessed, but paternal acceptance and rejection perceptions were not assessed.

### Clinical implications

ADHD is a prevalent psychiatric disorder, and it may cause significant complications if left untreated. The comorbidity of aggression has a negative influence on the treatment and prognosis of ADHD. In cases of ADHD comorbid with aggression, aggressive symptoms are more apparent and continuous compared to ADHD cases without aggression. Within this context, it is appropriate to evaluate ADHD cases first in terms of family factors, and then for cognitive and parent–child relational factors before the emergence of aggressive symptoms.

### Key points

• What’s known: Past research has shown that when a child is referred with aggressive symptoms, one of the most common diagnoses is attention-deficit hyperactivity disorder (ADHD).

• What’s new: Previous studies have not examined which demographic factors, family factors, perception of acceptance/rejection by the mothers and cognitive factors differentially correlate with aggression at home and at school.

• Findings: Family factors, cognitive factors and perception of acceptance/rejection by the mothers are important aspects of ADHD children’s aggression.

• This study confirms that family factors affect aggressive behaviors of ADHD children at home and at school settings.

• Cognitive factors determine the aggressive behaviors of elementary school students’ aggression in both school and home.

• The child’s perception of acceptance of rejection by the mothers is related to aggression at home and not to aggression at school.

• Implications: Prevention and intervention programs that target aggressive behaviors of ADHD children by focusing on family factors, cognitive factors and perception of acceptance rejection by parents may have the most impact.

## Competing interest

The study was not supported by any financial funding. No financial or material support was taken for the study. Dr. Ercan is on advisory boards for Eli Lilly Turkey and Janssen Turkey. The other authors have no biomedical financial interests or potential conflicts of interest.

## Authors’ contributions

All authors but BKB contributed equally to the design and conduct of the study, interpretation of the results, and writing of the manuscript. BKB was responsible for collection of the data. All authors read and approved the final manuscript.
